# Editorial: The role of oxidative stress and systemic inflammation in diabetes and chronic kidney disease

**DOI:** 10.3389/fendo.2023.1272525

**Published:** 2023-08-21

**Authors:** Dipanjan Chanda, Hunjoo Ha, In-Kyu Lee

**Affiliations:** ^1^ Research Institute of Aging and Metabolism, Daegu, Republic of Korea; ^2^ Graduate School of Pharmacy, College of Pharmacy, Seoul, Republic of Korea

**Keywords:** oxidative stress, inflammation, diabetes, chronic kidney disease, pathology

Oxidative stress and systemic inflammation are interconnected processes that can influence and amplify each other, leading to various diseases and health conditions. Oxidative stress can activate immune cells, such as macrophages, neutrophils, and monocytes, to release pro-inflammatory molecules. These activated immune cells can generate reactive oxygen species (ROS) as part of their defense mechanisms. Conversely, ROS can stimulate the production of pro-inflammatory cytokines, such as interleukin-6 (IL-6) and tumor necrosis factor-alpha (TNF-α). These cytokines, in turn, can trigger the generation of more ROS, creating a positive feedback loop between oxidative stress and inflammation. This loop can sustain chronic inflammation and lead to tissue damage in various organs. Many risk factors for oxidative stress and systemic inflammation overlap. Obesity, which is associated with chronic inflammation, is also linked to oxidative stress due to increased metabolic activity. Unsurprisingly, the relationship between oxidative stress and systemic inflammation is complex and bidirectional. They can synergistically contribute to the development and progression of various chronic diseases, including cardiovascular diseases, diabetes, neurodegenerative disorders, and certain cancers. Addressing lifestyle factors that contribute to oxidative stress and inflammation is essential in maintaining overall health and reducing the risk of these chronic conditions.

This Research Topic aimed to and highlighted our current understanding of this bidirectional relationship between inflammation and oxidative stress and their role in the pathophysiology and treatment of diabetes and CKD.

In an in-depth review (Wei et al.), the authors have focused on the downstream targets of Hypoxia-inducible factor-1 α (HIF-1α), a key transcriptional regulator that senses cellular oxygen status and plays a critical role in the pathogenesis of renal fibrosis. The authors have detailed the molecular underpinnings associated with HIF-1α-induced regulation in this context and shed light on potential therapeutic avenues to counter or ameliorate renal fibrosis.

Diabetic nephropathy (DN), a major microvascular abnormality of diabetes mellitus and a common cause of chronic renal failure among diabetic patients, has significant long-term impacts on the morbidity and mortality of diabetes patients. Although the exact mechanism underlying this complication is currently unknown, in this review (Wang et al.), the authors have tried to explore and delineate the current understanding of the role of Chinese herbal medicine (CHM) as a therapeutic option in alleviating this debilitating condition. CHM exhibits strong anti-inflammatory properties and has been shown to ameliorate albuminuria in diabetic patients. In this review, the authors have proposed a potential mechanistic explanation underlying the positive effects of CHM on DN, providing indications that DN therapy might be on the horizon.

Exploring the bidirectional relationship between microvascular and macrovascular complications in diabetes and elucidating the pathogenesis is key to prolonged life span of diabetic patients as well as to improving the quality of life. To address that, in the original research article (Yuchen et al.), the authors performed data mining and analysis of expression profiles from Gene Expression Omnibus database of human cohorts with DN, diabetic retinopathy (DR), atherosclerosis. This study concludes that chronic inflammation due to endothelial cell activation and oxidative stress is the common denominator linking atherosclerosis, diabetes retinopathy and diabetes nephropathy. Findings from this study can be of clinical and social significance in early detection and as well as in reducing the incidence of disabling or fatal circulatory complications in diabetes.

Diabetic peripheral neuropathy (DPN), a common complication in diabetic patients, is characterized by the presence of symptoms and/or signs of peripheral nerve dysfunction. Recent developments in this field of research have shed light on the abnormalities in programmed cell death to play a causative in the development of DPN. In this first of its kind bioinformatics-based study (Tian et al.), the authors have used data mining and analysis of differentially expressed genes from different datasets and intersected them with ferroptosis dataset to predict key genes, molecules and miRNAs that are involved in the pathogenesis of DPN. This study provides interesting insight and clues for future investigations about the role of ferroptosis in the development of DPN.

In another insightful research article (Qin et al.), the authors have utilized machine-learning (ML) models based on ultrasound radiomics for noninvasive evaluation of the crescent status in immunoglobulin A (IgA) nephropathy. This study concludes that ML classifier can be potentially valuable for noninvasive diagnosis of IgA nephropathy and help clinicians in selecting treatment strategies for nephropathy.

In conclusion, oxidative stress and systemic inflammation play significant roles in the development and progression of both diabetes and chronic kidney disease (CKD). Several mechanisms contribute to the link between diabetes and oxidative stress, including disrupted glucose metabolism, mitochondrial dysfunction, inflammation etc. leading to exacerbation of diabetic complications. Overall, oxidative stress and systemic inflammation are closely interconnected and contribute to the development and progression of both diabetes and CKD. Managing these processes through lifestyle modifications, antioxidant therapy, anti-inflammatory drugs, and proper management of diabetes and blood pressure can help mitigate complications and improve outcomes in individuals with these conditions.

## Author contributions

DC: Writing – original draft, Writing – review & editing. HH: Writing – review & editing. I-KL: Writing – original draft, Writing – review & editing.

